# Cook County Hospital

**Published:** 1875-11

**Authors:** D. A. K. Steele


### Hospitals.

### COOK COUNTY HOSPITAL.

(From Notes by D. A. K. Steele, M.D.)

### CANCER OF PYLORUS.

Catherine Y., a Polish washerwoman, aged 40 years, was admitted to the wards of the County Hospital, Feb. 2, 1874, stating that she had enjoyed good health until about five months previously, when she left Prussia to come to America. When at sea two weeks, she was severely attacked with sea sickness, vomiting all ingesta, even water. The matter ejected was of a greenish color. She was sick about three weeks ; partially regained her health after landing, but ever since the stomach has been irritable, and she is subject to attacks of vomiting.

On admission, patient poorly nourished and emaciated ; no appetite ; bowels constipated ; complains of pain in the chest, stomach and bowels ; has some difficulty in urinating ; frequently ejects a greenish-colored vomit ; has occasional rigors and fainting fits.

Physical exploration of the chest reveals no particular abnormality ; respiration is short, quick, jerky, and rather feeble ; pulse 88 ; respiration, 28. On examination, find the abdomen doughy, bowels gurgling on palpation ; find a hard nodulated mass just below the ensiform cartilage, extending on either side of the median line ; the mass being very tender on pressure. Patient was given wine, beef tea and eggs, *ad libitum*, and ordered a pill containing extract of conium, gr. ij ; ferric arseniate, gr. $\frac{1}{12}$, every four hours, and carbolic acid, gr. $\frac{1}{4}$, in mucilage, to control vomiting.

April 10. On examination, find cancerous deposit involving rectum ; abdominal cavity contains a large amount of fluid. Patient continued gradually growing worse until May 9th, when she died. Autopsy, seventeen hours after death. Body very much emaciated; considerable serum in abdominal cavity; peritoneum rough, and studded with irregular points of cancerous deposit, giving it a peculiar, mottled, pearly appearance; liver and spleen contained small masses of cancerous deposit. The pyloric orifice of the stomach was very much thickened, and the orifice contracted to about one-half its normal calibre; the walls of the pylorus being about three-fourths of an inch thick. Throughout the entire length of the intestinal tube, there were points of cancerous deposit, giving the intestines a roughened, granular, whitish or mottled appearance, resembling the spots on a rattlesnake. Along the left side of the rectum and lower part of the ileum, there were twelve or fourteen small cancerous masses, about the size of kernels of corn, and perfectly white; rectum was thickened. Lungs and heart seemingly healthy. The kidneys contained small deposits of cheesy matter. Brain not examined.

